# Diagnostic Efficacy and Clinical Impact of Image-guided Core Needle Biopsy of Suspected Adult Nonvertebral Osteomyelitis

**DOI:** 10.1093/ofid/ofaf665

**Published:** 2025-10-29

**Authors:** Winston L Winkler, Ige A Geroge, Sumanth Gandra, Jonathan C Baker, Anderanik Tomasian, Benjamin Northrup, Theodore L Vander Velde, Travis J Hillen, Chongliang Luo, Resten Imaoka, Gino M Dettorre, Jack W Jennings

**Affiliations:** Department of Radiology, Section of Musculoskeletal Radiology, Mallinckrodt Institute of Radiology, St. Louis, Missouri, USA; John T. Milliken Department of Internal Medicine, Division of Infectious Disease, Washington University in St. Louis School of Medicine, St. Louis, Missouri, USA; John T. Milliken Department of Internal Medicine, Division of Infectious Disease, Washington University in St. Louis School of Medicine, St. Louis, Missouri, USA; Department of Radiology, Section of Musculoskeletal Radiology, Mallinckrodt Institute of Radiology, St. Louis, Missouri, USA; Department of Radiology, Section of Musculoskeletal Radiology, University of Southern California, Los Angeles, California, USA; Department of Radiology, Section of Musculoskeletal Radiology, Mallinckrodt Institute of Radiology, St. Louis, Missouri, USA; Department of Radiology, Section of Musculoskeletal Radiology, Mallinckrodt Institute of Radiology, St. Louis, Missouri, USA; Department of Radiology, Section of Musculoskeletal Radiology, Mallinckrodt Institute of Radiology, St. Louis, Missouri, USA; Division of Public Health Sciences, Washington University School of Medicine in St. Louis, St. Louis, Missouri, USA; Department of Radiology, Section of Musculoskeletal Radiology, Mallinckrodt Institute of Radiology, St. Louis, Missouri, USA; Department of Radiology, Section of Musculoskeletal Radiology, Mallinckrodt Institute of Radiology, St. Louis, Missouri, USA; Department of Radiology, Section of Musculoskeletal Radiology, Mallinckrodt Institute of Radiology, St. Louis, Missouri, USA

**Keywords:** bone biopsy, diagnostic yield, image-guided core needle biopsy, non-vertebral osteomyelitis

## Abstract

**Objectives:**

The diagnostic yield and clinical impact of image-guided core needle biopsy (ICNB) of suspected nonvertebral osteomyelitis in adults is heterogenous in published studies because of small sample size, indicating the need for large cohort studies.

**Methods:**

A retrospective analysis of ICNBs was performed from 2010 to 2021 for patients with suspected nonvertebral osteomyelitis. For each biopsy, a series of factors were analyzed, as well as if histopathology was diagnostic of osteomyelitis and if microbiological cultures were positive. Additionally, it was recorded in what way biopsy influenced clinical management regarding antimicrobial treatment. Multivariate statistical analysis was performed to evaluate the factors associated with yield.

**Results:**

A total of 883 biopsies performed on 787 patients were included. A histopathologic diagnosis of osteomyelitis was made in 51.6% (381/738) of biopsies, and microbiological cultures were positive in 28.7% (253/883) of biopsies. Antimicrobial exposure before biopsy was negatively associated with positive cultures from bone core samples (odds ratio [OR] = 0.52; 95% confidence interval [CI], .33–.83; *P* = .0005). Elevated hemoglobin A1c (continuous variable) (OR = 1.38; 95% CI, 1.03–1.86; *P* = .03), and purulent aspirate (OR = 28.1; 95% CI, 2.67–1.86; *P* = .03) were positively associated with positive cultures from aspirate samples. Clinical management was affected by ICNB in 26.2% (231/883) of cases.

**Conclusions:**

In this large cohort, ICNB yielded approximately 30% positive cultures and changed clinical management in more than one fourth of patients.

**Summary Statement:**

In a retrospective study of 883 image-guided biopsies of suspected nonvertebral osteomyelitis, microbiological cultures were positive in 28.7% (253/883) of biopsies, a histopathologic diagnosis of osteomyelitis was made in 51.6% (197/381), and 26.2% (231/883) of biopsies affected clinical management.

Osteomyelitis diagnosis and etiologic identification is best accomplished by surgical sampling or by image-guided core needle biopsy (ICNB) and/or aspiration to obtain tissue for histopathologic examination and microbiological cultures [1, 2]. The identification and antimicrobial susceptibility data of a causative microorganism is crucial, as the current standard of care is to treat patients with at least 6 weeks of antimicrobials, along with surgical therapy whenever possible [3, 4].

Open surgical biopsy results in maximal tissue acquisition and is considered the reference standard for biopsy sampling. High rates of culture positivity have been reported for open biopsy of nonvertebral osteomyelitis, with estimates ranging from 75% to 93% [5–7]. ICNB, however, has become an increasingly common procedure because of lower cost, shorter hospitalization times, and a lower complication rate than open biopsy [8–10]. The role of ICNB of vertebral osteomyelitis is well established and is recommended by current Infectious Disease Society of America (IDSA) guidelines [11]. The utility of ICNB for adult nonvertebral osteomyelitis, however, is less clear. For diabetic pedal osteomyelitis, IDSA guidelines make a weak recommendation to perform ICNB only if there is diagnostic uncertainty or failure of response to empiric treatment and the patient is not undergoing surgery [4]. There are no guidelines for diagnosis and management of adult nonvertebral osteomyelitis in general. Furthermore, estimates for diagnostic yield of ICNB of nonvertebral osteomyelitis vary widely in published studies because of small sample sizes [12–20]. Moreover, few studies have evaluated how ICNB influences clinical management in regard to antimicrobial treatment [15, 16, 19]. The purpose of this study is to examine the diagnostic yield of ICNB of nonvertebral osteomyelitis in adults and evaluate which factors are associated with these results using the largest cohort (to our knowledge) of patients published to date. We then analyze if, and in what way, clinical management was changed by each biopsy.

## MATERIALS AND METHODS

A retrospective analysis of ICNBs was performed from 2010 to 2021 for patients with suspected nonvertebral osteomyelitis. Institution review board approval was obtained for this Health Insurance Portability and Accountability Act–compliant retrospective study. Consent was waived for retrospective participation and data analysis. All consecutive patients (as many cases as possible were included to maximize accuracy and power of the study) who received an ICNB at a single academic institution from July 2010 to July 2021 were identified using the radiology information system. At our academic medical center, clinicians, based on patient symptoms, physical exam, laboratory values, and imaging findings (such as bone marrow edema, erosive changes, periosteal reaction) produced by radiology reports would refer the patients to bone biopsy. The musculoskeletal radiologist then made the final decision regarding whether the entire picture (including imaging findings and clinical context) fit that of nonvertebral osteomyelitis and whether to perform the percutaneous biopsy. Patients who received the biopsy for sampling of suspected noninfectious lesions, suspected vertebral osteomyelitis, were younger than 18 years of age, or were lost to follow up, were excluded, leaving only adult patients who underwent biopsy for sampling of suspected nonvertebral osteomyelitis to be included.

For each biopsy a series of clinical, lesion-related, and technical factors were obtained from the electronic medical record (Supplementary table 1).

Primary outcomes included whether bone cores from biopsy were histologically sufficient for diagnosis and resulted in a histopathologic diagnosis of osteomyelitis. Also recorded were whether bone cores or aspirate samples sent for culture were positive and what the isolated microorganism(s) were. Microorganisms considered contaminants were excluded. This was at the discretion of the treating infectious disease attending, who made this designation based on the clinical context and considerations such as patient immunocompromised status, concurrent bacteremia, and suspected surgical hardware infection (if recorded). The electronic medical record was then reviewed for each patient's workup of osteomyelitis to determine how and in what way each biopsy influenced clinical management in regard to antimicrobial treatment for each patient. A change in management was considered to be present when the treating clinician referenced new information obtained from the bone biopsy that resulted in a decision to initiate, change, discontinue, or withhold antimicrobial treatment.

Computed tomography (CT) guidance was used when increased characterization of smaller lesions and better visualization of anatomy was desired, such as in the pelvis or sacrum. CT also allowed operators to more easily use anatomic landmarks seen on previous magnetic resonance imaging studies during biopsy of CT-occult lesions. Otherwise, fluoroscopy was generally used because it afforded increased procedure efficiency and relative decrease in ionizing radiation exposure.

Greater than 90% of the biopsies were performed using a coaxial technique with the OnControl Needle system (Teleflex, Wayne Pennsylvania), with aspiration attempted through the outer cannula for each biopsy. Additional details regarding sample processing and culture technique can be found in Supplementary Appendix 1. There was no independent review of the pathology results.

Multivariate logistic regression analysis was performed to analyze variables (Supplementary table 1) associated with histopathology being diagnostic of osteomyelitis, and positive microbiological cultures using R statistical software (version 4.1.3, 2022, opensource). Comparison *P* values were obtained by the chi-square test for categorical variables and the *t*-test for continuous variables. Variables were first tested in univariate analysis, and after backward stepwise selection, statistically significant factors (*P* < .05) were then subsequently tested in multivariate analysis. For continuous values, the odds ratio (OR) represents the odds that a value produces an outcome compared to one unit less than that value.

## RESULTS

### Patient Selection/Demographics

**Figure 1.**
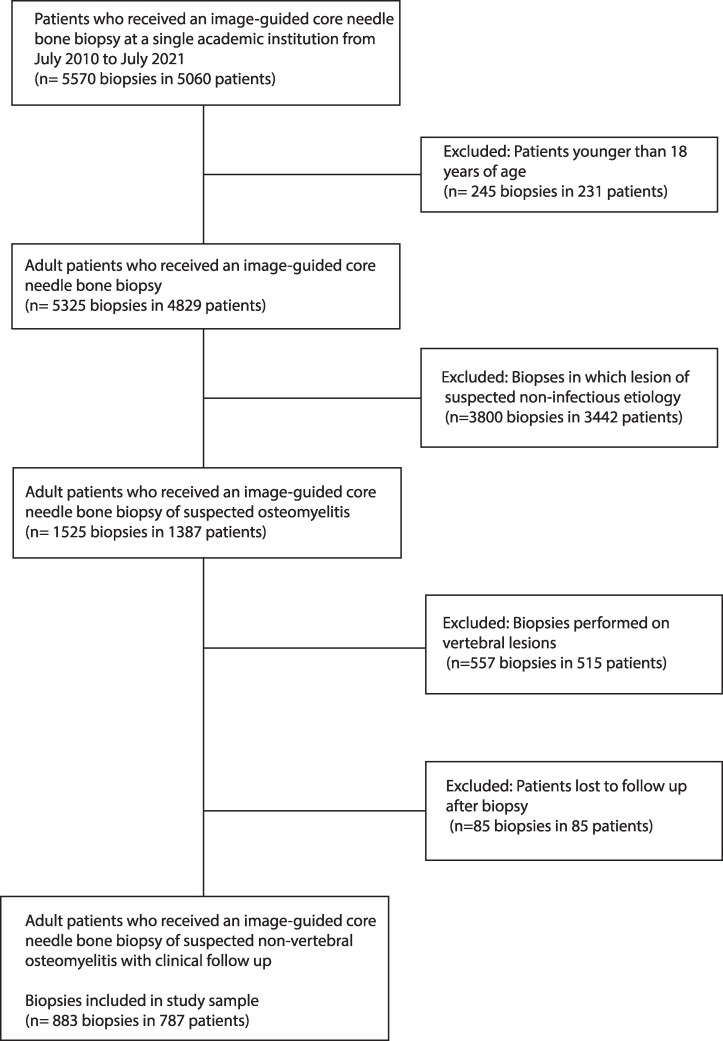
Study selection.

Overall, 5570 image-guided core needle bone biopsies were performed on 5060 patients between July 2010 and July 2021. Among 5570 biopsies, 245 biopsies performed on 231 patients were excluded because the patients were younger than 18 years of age, and 3800 biopsies performed on 3442 patients were excluded because the lesions were of noninfectious etiology. Subsequently, 557 biopsies performed on 515 patients were excluded because the biopsies were performed on vertebral lesions, and 85 biopsies performed on 85 patients were excluded because the patients were lost to follow up after biopsy. This subsequently left 883 biopsies performed on 787 patients as the study sample (Figure 1). Demographic characteristics are detailed in Table 1.

**Table 1.** Patient Demographic Characteristics, as Well as Clinical, Lesion-related, and Technical Factors of All Biopsies (Including Rebiopsies)

Factor	Total Biopsies	Biopsies With Positive Cultures (Either Bone Core or Aspirate Samples)	Biopsies With Negative Cultures
Mean patient age, years	54.35 ± 16.0 (standard deviation)	55.1 ± 16.2	54.1 ± 15.9
Males	589 (66.7%)	170 (67.2%)	419 (66.5%)
Diabetes	442 (50.1%)	139 (54.9%)	303 (48.1%)
Peripheral vascular disease	132 (14.9%)	45 (17.8%)	87 (13.8%)
Immunocompromised patients	96 (10.9%)	28 (11.1%)	68 (10.8%)
Smoking history (any)	481 (54.5%)	143 (56.5%)	338 (53.7%)
Suspected acute osteomyelitis	278 (31.5%)	108 (38.9%)	170 (61.2%)
Suspected chronic osteomyelitis	605 (68.5%)	145 (24.0%)	460 (76.0%)
Number of biopsies in which antibiotics were administered within 2 weeks before biopsy	485 (54.9%)	127 (50.2%)	358 (56.8%)
Number of biopsies in which patient had history of fever before biopsy	201 (22.8%)	62 (24.5%)	139 (22.1%)
Number of biopsies in which patient had positive blood cultures	38 (4.3%)	22 (8.7%)	16 (2.5%)
Number of biopsies in which patient had elevated WBC count	263 (29.8%)	83 (32.8%)	180 (28.6%)
Mean WBC count (×10^9^/L) for all patients	8.9 ± 4.7	9.1 ± 3.8	8.9 ± 5.0
Number of biopsies in which patient had elevated ESR	657 (74.4%)	203 (80.2%)	454 (72.1%)
Mean ESR (mm/h) for all patients	60.6 ± 34.3	62.3 ± 32.9	59.9 ± 34.8
Mean HbA1c (%) for all patients	7.8 ± 2.4	8.3 ± 2.5	7.6 ± 2.3

Abbreviations: ESR, erythrocyte sedimentation rate; HbA1C, hemoglobin A1C; WBC, white blood cell.

Details of image findings, procedural details, procedural yield, antimicrobial exposure, and other relevant laboratory values are reported in Table 1 and Supplementary Table 2. Four hundred and eighty-eight patients (54.9%) had antimicrobial exposure within 2 weeks before ICBN. Of these, 414 (85.4%) received antimicrobials until the day of biopsy.

### Biopsy Diagnostic Efficacy

Of the 787 biopsies in which bone cores were sent for histopathological analysis, specimens were histologically adequate and sufficient for diagnosis in 93.8% (738/787) of biopsies. A histopathological diagnosis of osteomyelitis was made in 51.6% (381/738) of total biopsies. Of these 381 biopsies in which osteomyelitis was histopathologically confirmed, cultures were positive in 141 (37.0%). For these biopsies, 51.7% (197/381) had previous antimicrobial exposure. In multivariate analysis elevated erythrocyte sedimentation rate (ESR; vs normal range) (OR = 1.66; 95% confidence interval [CI], 1.05–2.63; *P* = .03), was positively associated with histopathology being diagnostic of osteomyelitis. Lesion location in the foot was negatively associated with histopathology diagnostic of osteomyelitis (OR = 0.72; 95% CI, .51–1.01; *P* = .052) but this association was not found to be statistically significant.

Overall, cultures from either bone cores or collected aspirate were positive in 28.7% (253/883) of biopsies. Cultures from bone cores were positive in 24.2% (199/823) of biopsies and cultures from aspirate samples were positive in 25.7% (54/210). Gram-positive organisms were the most frequent organisms, with coagulase negative *Staphylococcus* being the most common. *Pseudomonas aeruginosa* was the most common gram-negative organism (Table 2). Microorganisms isolated from culture and their frequencies can be found in Table 2 and Supplementary Table 3. In multivariable analysis, increasing age (OR = 1.02; 95% CI, 1.01–1.03; *P* = .02) was positively associated with positive cultures from bone core samples, whereas antimicrobial exposure before biopsy was negatively associated with positive cultures from bone core samples (OR = 0.52, 95% CI, .33–.83; *P* < .001) (Table 3). For aspirate samples, elevated hemoglobin A1c (continuous variable) (OR = 1.38; 95% CI, 1.03–1.86; *P* = .03) and purulent aspirate (OR = 28.1, 95% CI, 2.67–1.86; *P* = .03) were associated with positive cultures (Table 3).

**Table 2.** Microorganisms Isolated From Culture and Their Frequency for All Biopsies

Microorganism	Frequency Isolated From Bone Core/Aspiration Samples
Gram-positive	
Coagulase negative *Staphylococci*	64 (21.3%)
MRSA	23 (7.6%)
MSSA	42 (14.0%)
*Streptococcus* species	31 (10.3%)
Other gram-positive	54 (17.9%)
Gram-negative	
*Pseudomonas aeruginosa*	19 (6.3%)
*Proteus mirabilis*	13 (4.3%)
*Escherichia coli*	10 (3.3%)
Other gram-negative	19 (6.3%)
Fungal organisms	
*Candida* species	6 (2.0%)
Others	3 (1.0%)
Polymicrobial not specified	15 (5.0%)
*Mycobacterium* species	2 (0.7%)

In 53 cases with positive culture, more than 1 organism was isolated.

Abbreviations: MRSA, methicillin-resistant Staphylococcus aureus; MSSA, methicillin-sensitive Staphylococcus aureus.

**Table 3.** Multivariate Statistical Analysis for Factors Selected by Backward Stepwise Selection for All Biopsies

Factors Associated With Bone Cores Being Histopathologically Diagnostic of Osteomyelitis	Odds Ratio	95% Confidence Interval	*P* Value
Elevated ESR	1.67	1.05–2.64	.03
Location in foot	0.72	.51–1.01	.052
Factors associated with positive cultures from bone core			
Increasing age	1.02	1.003–1.03	.02
Any antimicrobial exposure 2 weeks before biopsy	0.52	.33–.826	.005
Factors associated with positive cultures from aspirate			
Elevated hemoglobin A1c	1.38	1.03–1.86	.03
Purulent aspirate	28.1	2.67–296.37	.005

Abbreviation: ESR, erythrocyte sedimentation rate.

### Change in Management

Biopsy results influenced clinical management in 26.2% (231/883) of patient cases. In 150 cases (17.0% of total biopsies), cultures were positive and antimicrobial treatment was tailored to culture results. In 49 patient cases (5.5% of total biopsies), antimicrobials were discontinued or not initiated because of negative cultures and histopathology. For these cases, information was not available in regard to alternate diagnoses which explained the patient's presentation. In 20 cases (2.3%), histopathology was diagnostic of chronic osteomyelitis, cultures were negative, and the patient was clinically improving/asymptomatic and antimicrobials were thus not initiated (biopsy was pursed in these cases to exclude alternate diagnoses that mimic chronic osteomyelitis on imaging, such as osseous neoplasms). In 4 cases (0.5%), empiric antimicrobials were initiated because of a histopathologic diagnosis of osteomyelitis, in the setting of negative cultures. In 5 cases (0.6%), empiric treatment was broadened because of the polymicrobial result of cultures. In 3 cases (0.3%), a diagnosis of gout was made, and antimicrobials were not initiated or discontinued.

**Figure 2.**
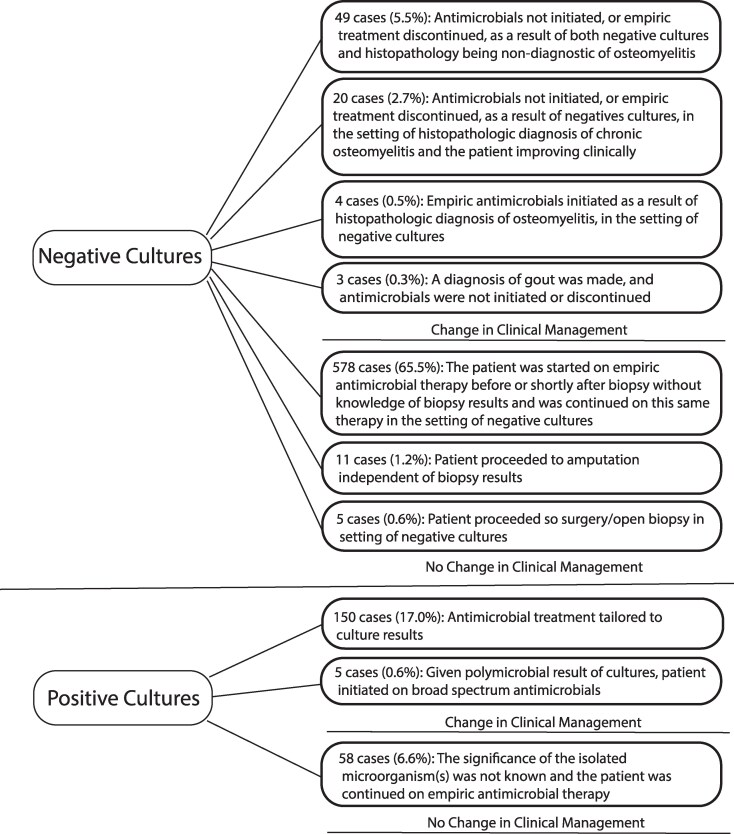
Change in clinical management by culture results following image-guided core needle biopsy among 883 biopsies performed on 787 patients with suspected nonvertebral osteomyelitis between 2010 and 2021.

Biopsy did not change clinical management in 73.8% (652/883) of patient cases (Figure 2). In 578 cases (65.5% of total biopsies), the patient was started on empiric antimicrobial therapy before or shortly after biopsy without knowledge of biopsy results and was continued on this same therapy in the setting of negative cultures. In 40.0% (231/578) of these cases, histopathology was diagnostic of osteomyelitis (108 cases of chronic osteomyelitis, 123 acute) in 45.7% (264/578) histopathology was not diagnostic of osteomyelitis, and in 14.4% (83/578) histopathology was not obtained or was insufficient for diagnosis. Thus, in 39.3% (347/883) of total biopsies, there was no histopathologic evidence of osteomyelitis and cultures were negative but empiric antimicrobials were continued regardless (in the setting of presumed false-negative biopsy results). In 58 (6.6%) cases, cultures were positive, but the significance of the isolated organism was not known (without it being considered a contaminant) and the patient was continued on empiric antimicrobial therapy. In 11 cases (1.2%), the patient proceeded to amputation independent of biopsy results. In 5 cases (0.6%), the patient proceeded to open biopsy/surgery after cultures were negative.

In 55 cases, histopathology was negative for a diagnosis of osteomyelitis but cultures were positive, usually in the setting of histopathology yielding either nonspecific fibrosis or osteonecrosis. In these cases, the histopathology was regarded as falsely negative, and the clinical and imaging findings supporting osteomyelitis along with the positive cultures was considered sufficient to make a diagnosis of osteomyelitis.

### Complications

Complications were documented in 0.1% (1/883) biopsies. In this case, the patient experienced gluteal hematoma after a sacral biopsy.

## DISCUSSION

In the large cohort examined in our study, a histopathologic diagnosis of osteomyelitis was made in 51.6% (381/738) of biopsies, microbiological cultures were positive in 28.7% (253/883), and clinical management was affected in 26.2% (231/883) of patient cases. We observed that elevated ESR was positively associated with histopathological diagnosis of osteomyelitis. Antimicrobial exposure within 2 weeks was associated with negative bone cultures, whereas increasing age was associated with positive bone culture results. For aspirate cultures, purulent fluid and elevated hemoglobin A1c were associated with positive cultures.

Overall, the results of our study are in accordance with other studies in literature. Microbiological culture positivity rates have previously been reported to range from 21% to 69%, whereas rates of positive histopathologic sampling vary between 29% and 52% [12–20]. Additionally, rates in which ICNB changed clinical management have been reported to range between 5% and 48% [12–20]. A recently published meta-analysis by Smayra et al. examined the yield and clinical impact of ICNB of the appendicular skeleton [20]. This study included 8 total studies; however, 2 of these studies were of exclusively pediatric patients. If the pediatric studies are excluded, then the pooled culture positivity rate reported by Smayra et al. is 26.7% (193/722), the rate at which a histopathologic diagnosis of osteomyelitis was made is 47.1% (173/367), and the rate of postprocedural change in management is 42.2% (212/502). The diagnostic yield reported by this meta-analysis is concordant with the results of our study; however, the postprocedural change in management rate is substantially higher. In this meta-analysis, however, a change in management was counted any time there was any modification in antibiotics. This differs from our study, in which a change in management was considered to be present only when the treating clinician referenced new information obtained from the bone biopsy that resulted in antimicrobial modification. Thus, it is possible that the meta-analysis overestimated the rate of change in management if antibiotics were modified due to factors independent of the biopsy results, such as patient medical comorbidities, or adverse drug effects.

The overall role of ICNB of nonvertebral osteomyelitis is less established than for vertebral osteomyelitis. For vertebral osteomyelitis, current IDSA guidelines recommend ICNB when a previous microbiologic diagnosis has not already been established through blood cultures or serology [10]. There are no similar guidelines for the diagnosis and management of adult nonvertebral osteomyelitis in general. Given that ICNB yielded a diagnosis of osteomyelitis in 51.6% of biopsies, changed clinical management in 26.2% of cases, and resulted in very few complications, we overall feel that the present study justifies the more routine use of ICNB for suspected nonvertebral osteomyelitis, similar to the role of ICNB in vertebral osteomyelitis. In this study, for 25.1% of patients (222/883), antibiotics were either tailored or discontinued because of the results of ICNB. Thus, ICNB spared a signification fraction of patients from the potential adverse effects of broad empiric antibiotic treatment and meaningfully contributed to antibiotic stewardship during a time of increasing concern for antimicrobial resistance. It is important to note, however, that ICNB for nonvertebral lesions might not be as effective as for vertebral lesions. In our study examining the diagnostic efficacy of ICNB in vertebral osteomyelitis, ICNB resulted in a histopathologic diagnosis of osteomyelitis in 68.4%, 29.6% positive cultures, and changed management in 37.5% of patient cases [21]. Although speculative, ICNB of vertebral lesions might have higher diagnostic yield because the rich vascular supply of the vertebrae might lead to greater microbiological seeding in cases of hematogenous osteomyelitis. Moreover, it is important to note that in 39.3% (347/883) of total biopsies, there was no histopathologic evidence of osteomyelitis and cultures were negative, but empiric antimicrobials were continued regardless. In these cases, antimicrobials were continued in the setting of presumed false-negative biopsy results given the imaging and clinical findings supported a diagnosis of osteomyelitis. Thus, ICNB was largely treated as an insensitive test, in which negative results did not rule out a diagnosis, but positive results usually led to clinically actionable data that changed clinical management. It is important to note however that antimicrobials were administered within 2 weeks of biopsy in 65.1% (226/347) of these cases, which might have increased the proportion of negative results in our cohort. Further research examining the sensitivity of ICNB for adult nonvertebral osteomyelitis is warranted.

In our study, antimicrobial exposure was associated with decreased culture positivity rates from bone core samples. Several other studies have examined if antimicrobial exposure is associated with positive cultures, and these studies found no association [7, 13, 15, 17]. It is possible, however, that these studies were under powered to detect such a difference, as the sample size of these studies were all relatively small (31–203 biopsies). Unlike vertebral osteomyelitis, in which it is recommended to withhold antibiotics until a microbiologic diagnosis is established, there are no such recommendations for the diagnosis and management of adult nonvertebral osteomyelitis [11]. Considering that in our study antimicrobial exposure was negatively associated with positive cultures from bone cores (with an OR of 0.52, *P* = .0005), our data demonstrate the importance of withholding antimicrobials before biopsy if possible.

Knowledge of the factors associated with positive microbiological results might help identify and select patients in whom ICNB would be most useful. In our study, aside from the effect of antimicrobials as explained earlier, increasing age was associated with positive cultures from bone cores, and elevated hemoglobin A1c and purulent aspirate were associated with positive cultures from aspirate samples. Purulent aspirate, elevated ESR, and elevated hemoglobin A1c relate to the level of microbiological burden of a lesion, and it makes intuitive sense that these factors would be associated with increased yield. Wu et al. also found that purulent aspirate was associated with positive cultures from aspirate samples [13]. Hirschfield et al. found leukocytosis to be associated with positive cultures from bone cores but did not find elevated ESR or C-reactive protein to be associated [15]. It is unclear why increasing age is associated with positive bone culture. Interestingly, increasing age was associated with decreased yield of positive cultures for vertebral osteomyelitis [21].

The main limitation of the study is that it reflects the experience of only 1 academic institution, and thus demographics of the population, biopsy referral patterns, and antimicrobial prescribing patterns may not reflect the diversity of clinical practice. The retrospective nature of the study could have led to biases based on clinical circumstances that led to continuing or withholding of antibiotics before biopsy, which could have affected the outcomes. We also did not examine if susceptibility to previously administered antibiotics may have changed the significance of the findings. Additionally, it was not assessed if cases of nonvertebral osteomyelitis were associated with surgical hardware. History of trauma was also not assessed because, for a significant number of patients (especially in patients with diabetes), it was not documented in the medical record. Moreover, because the final decision of whether to pursue biopsy was based on the assessment of the musculoskeletal radiology attending, there is some inherent subjectivity to our study selection. Likewise, the interpretation of culture data, including the assessment of the pathogenicity of possible contaminants and the way in which to tailor antimicrobial treatment, was at the discretion of the infectious disease attending and also subject to an inherent level of subjectivity. Similarly, there was no independent review of pathology results, also resulting in some subjectivity. Last, the operator heterogeneity of the radiologists who performed the biopsies might have confounded results.

In conclusion, in our single-center large cohort of patients, image-guided biopsy of suspected nonvertebral osteomyelitis yielded positive cultures in close to 30% of biopsies and changed clinical management in more than one fourth of patients, while having an extremely low complication rate. Withholding antimicrobials before the biopsy when possible was the only modifiable factor associated with increased microbiological yield.

## Supplementary Material

ofaf665_Supplementary_Data

## Supplementary Data

Supplementary materials are available at *Open Forum Infectious Diseases* online. Consisting of data provided by the authors to benefit the reader, the posted materials are not copyedited and are the sole responsibility of the authors, so questions or comments should be addressed to the corresponding author.
