# Supplementary material for: Diagnostic Efficacy and Clinical Impact of Image-guided Core Needle Biopsy of Suspected Adult Nonvertebral Osteomyelitis
Source: Open Forum Infect Dis. 2025 Oct 29;12(11):ofaf665. doi: 10.1093/ofid/ofaf665 (PMC12628504; doi:10.1093/ofid/ofaf665)
Supplement: ofaf665_Supplementary_Data [file ofaf665_supplementary_data.zip › Supplemental Appendix 1 Major Revision.docx]

**Supplementary Appendix 1:**

**Culture Techniques**

Specimens were collected in anaerobic transport media (Anaerobe Systems Morgan Hill, CA, USA) and in a sterile container and processed by the microbiology laboratory round the clock. Bone and tissue specimens were ground to a homogeneous consistency using a disposable tissue grinder for bacterial and mycobacterial cultures. Specimens for fungal culture were processed by cutting into small pieces and gently embedded into the agar. Blood agar, chocolate agar, MacConkey agar were used for aerobic cultures while Brucella blood agar was used for anaerobic cultures. Brain heart infusion agar and inhibitory mold agar were used for fungal cultures. Lowenstein Jensen agar (solid media) and mycobacterial growth indicator tube (MGIT) broth (liquid media) were used for mycobacterial cultures. Aerobic media were held for five days in 5% CO2 at 35^0^C and anaerobic media were held for five days in anaerobic conditions at 35^0^C. Fungal cultures were held for 28 days in an air incubator at 30 degrees while mycobacterial cultures were held for 6weeks in BACTEC MGIT system (BD Diagnostics, Sparks, MD, USA) for liquid media and 8 weeks at 35^0^C in an air incubator for solid media. Broad range 16S ribosomal RNA gene polymerase chain reaction (PCR) was not utilized routinely as standard of care for pathogen detection at our institution.
