# Supplementary material for: Diagnostic Efficacy and Clinical Impact of Image-guided Core Needle Biopsy of Suspected Adult Nonvertebral Osteomyelitis
Source: Open Forum Infect Dis. 2025 Oct 29;12(11):ofaf665. doi: 10.1093/ofid/ofaf665 (PMC12628504; doi:10.1093/ofid/ofaf665)
Supplement: ofaf665_Supplementary_Data [file ofaf665_supplementary_data.zip › Supplemental Table 1.docx]

**Supplemental Table 1**: Patient demographic characteristics, as well as clinical, lesion-related, and technical factors recorded for each biopsy.

| Factor: |
| --- |
| Patient age |
| Patient sex |
| Lesion anatomic location (Foot, Lower extremity (excluding foot), Pelvis, Sacrum, Upper Extremity, Clavicle/Sternum/Ribs/Scapula) |
| Biopsy needle gauge |
| Number of bone cores obtained |
| Total biopsy core length obtained (sum length of all cores obtained, in millimeters) |
| Volume of aspirate if obtained (milliliters) |
| Description of aspirate (bloody or purulent) |
| Imaging modality used during biopsy (CT or fluoroscopy) |
| Radiation dose during biopsy |
| Type of sedation (general anesthesia, conscious sedation, no sedation) during biopsy |
| Total monitored sedation time |
| Doses of fentanyl and midazolam received |
| Procedure-related complications |
| If the patient had antimicrobial exposure within the two weeks prior to biopsy |
| If antimicrobials were intravenous or oral |
| If the patient was concurrently receiving antimicrobials or antimicrobials had been stopped within two weeks |
| Symptom duration (with suspected acute osteomyelitis defined as less than four, and chronic as greater than four weeks) ^22^ |
| Objective or subjective history of fever within two weeks prior to biopsy |
| Positive blood cultures |
| Elevated (from reference range) white blood cell count (anytime 90 days prior to biopsy) |
| Elevated ESR (anytime 90 days prior to biopsy) |
| Elevated CRP (anytime 90 days prior to biopsy) |
| Elevated hemoglobin A1c (anytime 90 days prior to biopsy) |
| Medical history of diabetes (type one or two) |
| Medical history of immunocompromised condition (diseases causing leukopenia or immunosuppressive medications) |
| Medical history of peripheral vascular disease |
| Sinus tract present during physical exam |
| Exposed bone present, or probe to bone test positive during physical exam |
| Ulcer greater than 2 cm present during physical exam |
| Medical history of peripheral vascular disease |
| Any lifetime smoking history |
