# Supplementary material for: Diagnostic Efficacy and Clinical Impact of Image-guided Core Needle Biopsy of Suspected Adult Nonvertebral Osteomyelitis
Source: Open Forum Infect Dis. 2025 Oct 29;12(11):ofaf665. doi: 10.1093/ofid/ofaf665 (PMC12628504; doi:10.1093/ofid/ofaf665)
Supplement: ofaf665_Supplementary_Data [file ofaf665_supplementary_data.zip › Supplemental Table 2.docx]

**Supplemental Table 2:** Clinical, Lesion-related, and Technical Factors of All Biopsies (Including Rebiopsies)

| Factor | Total Biopsies | Biopsies with positive cultures (either bone core or aspirate samples) | Biopsies with negative cultures |
| --- | --- | --- | --- |
| Anatomical location of lesions | Foot: 502 (56.9%)  Lower extremity (excluding foot): 108 (12.2%)  Pelvis: 129 (14.6%)  Sacrum: 92 (10.4%)  Upper Extremity: 36 (4.1%)  Clavicle/Sternum/Ribs/Scapula: 16 (1.8%) | Foot: 156 LE (61.6 %)  Lower extremity (excluding foot): 18 (7.1%)  Pelvis: 36 (14.2%)  Sacrum: 25 (9.9%)  Upper Extremity: 9 (3.6%)  Clavicle/Sternum/Ribs/Scapula: 9 (3.6%) | Foot: 346 LE (54.9 %)  Lower extremity (excluding foot): 90 (14.3 %)  Pelvis: 93 (14.8%)  Sacrum: 67 (10.6%)  Upper Extremity: 27 (4.3%)  Clavicle/Sternum/Ribs/Scapula: 7 (1.1%) |
| Mean biopsy needle gauge | 11.7 ± 1.2 | 11.6 ± 1.2 | 11.7 ± 1.2 |
| Mean number of bone cores sent for histopathologic analysis | 1.2 ± 0.5 | 1.2 ± 0.4 | 1.2 ± 0.5 |
| Mean total core length (mm) of biopsy sample sent for histopathologic analysis | 10.2 ± 5.8 | 11.0 ± 6.1 | 9.9 ± 5.4 |
| Mean number of bone cores sent for microbiological culture | 1.4 ± 0.7 | 1.4 ± 0.6 | 1.4 ± 0.7 |
| Mean total core length (mm) of biopsy sample sent for microbiological culture | 12.6 ± 8.8 | 12.8 ± 8.5 | 12.5 ± 9.0 |
| Mean aspirate volume obtained (mL) | 3.9 ± 4.5 | 4.1 ± 5.4 | 3.8 ± 4.2 |
| Number of aspirate samples with various descriptions | Bloody: 117 (55.5%)  Purulent: 17 (8.0%)  Not described: 77 (36.5 %) | Bloody: 14 (35.9%)  Purulent: 12 (30.7%)  Not described: 13 (33.3%) | Bloody: 103 (59.9%)  Purulent: 5 (2.9%)  Not described: 64 (37.2 %) |
| Number of biopsies performed under various imaging modalities | CT: 264 (29.9%)  Fluoroscopy: 619 (70.1%) | CT: 77 (30.4%)  Fluoroscopy: 176 (69.6%) | CT: 187 (29.7%)  Fluoroscopy: 443 (70.3%) |
| Mean CT radiation (mGy) | 353.0 ± 200.4 | 342.4 ± 148.5 | 357.8 ± 220.0 |
| Mean fluoroscopy radiation(mGy) | 83.6 ± 17.6 | 77.8 ± 14.4 | 86.3 ± 21.9 |
| Number of biopsies performed under various types of anesthesia/sedation | Conscious sedation- 421 (47.7%)  General anesthesia: 3 (0.3%)  No sedation: 459(52.0%) | Conscious sedation- 117 (46.2%)  General anesthesia: 0 (0.0%)  No sedation: 136(53.8%) | Conscious sedation- 304 (47.7%)  General anesthesia: 3 (0.3%)  No sedation: 323 (51.3%) |
| Number of biopsies with various types and timing of prior antibiotic administration | Concurrent IV antibiotics: 350 (72.2%),  Concurrent oral antibiotics: 64 (13.2%),  IV antibiotics stopped within two weeks of biopsy: 57 (11.8%)  Oral antibiotics stopped within two weeks of biopsy: 14 (2.9%) | Concurrent IV antibiotics: 81 (63.8%),  Concurrent oral antibiotics: 20( 15.7%),  IV antibiotics stopped within two weeks of biopsy: 22 (17.3%)  Oral antibiotics stopped within two weeks of biopsy: 4 (3.1%) | Concurrent IV antibiotics: 269 (75.1%),  Concurrent oral antibiotics: 44 (12.3%),  IV antibiotics stopped within two weeks of biopsy: 35 (9.8%)  Oral antibiotics stopped within two weeks of biopsy: 10 (2.8%) |
| Number of biopsies in which patient had elevated CRP levels | 369 (41.8%) | 114 (45.1%) | 255 (40.5%) |
| Mean CRP (mg/dl) level for all patients | 84.1 ± 84.3 | 91.6 ± 87.3 | 80.9 ± 83.0 |
| Number of biopsies in which patient had sinus tract present during physical exam | 10 (1.1%) | 1 (0.4%) | 9 (1.4%) |
| Number of biopsies in which patient had exposed bone present, or probe to bone test positive during physical exam | 65 (7.4%) | 18 (7.1%) | 47 (7.5%) |
| Number of biopsies in which patient had ulcer greater than 2 cm present during physical exam | 165 (18.7%) | 49 (19.4%) | 116 (18.4%) |
