# Supplementary material for: Diagnostic Efficacy and Clinical Impact of Image-guided Core Needle Biopsy of Suspected Adult Nonvertebral Osteomyelitis
Source: Open Forum Infect Dis. 2025 Oct 29;12(11):ofaf665. doi: 10.1093/ofid/ofaf665 (PMC12628504; doi:10.1093/ofid/ofaf665)
Supplement: ofaf665_Supplementary_Data [file ofaf665_supplementary_data.zip › Supplemental Table 3.docx]

**Supplemental Table 3**: Microorganisms Isolated from Culture and their Frequency for All Biopsies (Not listed in Table 2)

| Microorganisms | Frequency isolated from bone core/aspiration samples |
| --- | --- |
| **Gram-Positive** |  |
| *Corynebacterium* species | 20 |
| *Enterococcus faecalis* | 10 |
| *Actinomyces* species | 7 |
| *Cutibacterium acnes* | 6 |
| *Treperella* species | 2 |
| *Arcanobacterium haemolyticum* | 1 |
| *Clostridium ramosum* | 1 |
| *Enterococcus avium* | 1 |
| *Enterococcus faecium* | 1 |
| *Lactobacillus species* | 1 |
| *Micrococcus species* | 1 |
| *Parvimonas micra* | 1 |
| *Peptoniphilus asaccharolyticus* | 1 |
| *Vancomycin-resistant Enterococci* | 1 |
| **Gram-Negative** |  |
| *Bacteroides* species | 7 |
| *Klebsiella pneumoniae* | 3 |
| *Enterobacter cloacae* | 2 |
| *Alcaligenes faecalis* | 1 |
| *Campylobacter species* | 1 |
| *Haemophilus parainfluenzae* | 1 |
| *Prevotella bivia* | 1 |
| *Serratia marcescens* | 1 |
| *Stenotrophomonas maltophilia* | 1 |
| *Veillonella parvula* | 1 |
| **Fungal Organisms** |  |
| *Acremonium* species | 1 |
| *Aspergillus niger* | 1 |
| *Scedosporium apiospermum* | 1 |
